# Supplementary material for: Hip Surgery in Cerebral Palsy: A Bibliometric Analysis
Source: Int J Environ Res Public Health. 2023 Jan 18;20(3):1744. doi: 10.3390/ijerph20031744 (PMC9914051; doi:10.3390/ijerph20031744)
Supplement: Supplementary file 1 [file ijerph-20-01744-s001.zip › ijerph-2136672-supplementary.pdf]

## Supplemental Materials

Table S1. List of included studies

| First Author | Times Cited | Article Title | Country of origin | Journal | Impact factor 2021 | Publication Year |
|---|---|---|---|---|---|---|
| Hagglund | 181 | Prevention Of Dislocation Of The Hip In Children With Cerebral Palsy - The First Ten Years Of A Population-Based Prevention Programme | Sweden | Journal Of Bone And Joint Surgery-British Volume | 3.309 | 2005 |
| Hagglund | 136 | Prevention Of Dislocation Of The Hip In Children With Cerebral Palsy 20-Year Results Of A Population-Based Prevention Programme | Sweden | Bone & Joint Journal | 5.385 | 2014 |
| Dobson | 131 | Hip Surveillance In Children With Cerebral Palsy - Impact On The Surgical Management Of Spastic Hip Disease | Australia | Journal Of Bone And Joint Surgery-British Volume | 3.309 | 2002 |
| Ounpuu | 110 | Long-Term Effects Of Femoral Derotation Osteotomies: An Evaluation Using Three-Dimensional Gait Analysis | USA | Journal Of Pediatric Orthopaedics | 2.537 | 2002 |
| Kalen | 94 | Prevention Of Spastic Paralytic Dislocation Of The Hip | USA | Developmental Medicine And Child Neurology | 4.864 | 1985 |
| Bleck | 89 | The Hip In Cerebral-Palsy | USA | Orthopedic Clinics Of North America | 2.771 | 1980 |
| Sharrard | 87 | Surgical Prophylaxis Of Subluxation And Dislocation Of Hip In Cerebral-Palsy | England | Journal Of Bone And Joint Surgery-British Volume | 3.309 | 1975 |
| Miller | 79 | Reconstruction Of The Dysplastic Spastic Hip With Peri-Ilial Pelvic And Femoral Osteotomy Followed By Immediate Mobilization | USA | Journal Of Pediatric Orthopaedics | 2.537 | 1997 |
| Hoffer | 77 | Femoral Varus-Derotation Osteotomy In Spastic Cerebral-Palsy | USA | Journal Of Bone And Joint Surgery-American Volume | 6.558 | 1985 |
| Root | 73 | The Treatment Of The Painful Hip In Cerebral-Palsy By Total Hip-Replacement Or Hip Arthrodesis | USA | Journal Of Bone And Joint Surgery-American Volume | 6.558 | 1986 |
| Presedo | 71 | Soft-Tissue Releases To Treat Spastic Hip Subluxation In Children With Cerebral Palsy | USA | Journal Of Bone And Joint Surgery-American Volume | 6.558 | 2005 |
| Shore | 69 | Adductor Surgery To Prevent Hip Displacement In Children With Cerebral Palsy: The Predictive Role Of The Gross Motor Function Classification System | Australia | Journal Of Bone And Joint Surgery-American Volume | 6.558 | 2012 |
| Miller | 66 | Soft-Tissue Release For Spastic Hip Subluxation In Cerebral Palsy | USA | Journal Of Pediatric Orthopaedics | 2.537 | 1997 |

| | | | | | | |
|---|---|---|---|---|---|---|
| Silver | 63 | Adductor Release In Nonambulant Children With Cerebral-Palsy | Canada | Journal Of Pediatric Orthopaedics | 2.537 | 1985 |
| Dreher | 57 | Long-Term Outcome Of Femoral Derotation Osteotomy In Children With Spastic Diplegia | Germany | Gait & Posture | 2.746 | 2012 |
| Schwartz | 54 | Femoral Derotational Osteotomy: Surgical Indications And Outcomes In Children With Cerebral Palsy | USA | Gait & Posture | 2.746 | 2014 |
| Song | 54 | Femoral Varus Derotation Osteotomy With Or Without Acetabuloplasty For Unstable Hips In Cerebral Palsy | South Korea | Journal Of Pediatric Orthopaedics | 2.537 | 1998 |
| Stasikelis | 53 | Complications Of Osteotomies In Severe Cerebral Palsy | USA | Journal Of Pediatric Orthopaedics | 2.537 | 1999 |
| Widmann | 51 | Resection Arthroplasty Of The Hip For Patients With Cerebral Palsy: An Outcome Study | USA | Journal Of Pediatric Orthopaedics | 2.537 | 1999 |
| Sutherland | 51 | Psoas Release At The Pelvic Brim In Ambulatory Patients With Cerebral Palsy: Operative Technique And Functional Outcome | USA | Journal Of Pediatric Orthopaedics | 2.537 | 1997 |
| Saraph | 50 | Effect Of Derotation Osteotomy Of The Femur On Hip And Pelvis Rotations In Hemiplegic And Diplegic Children | Austria | Journal Of Pediatric Orthopaedics-Part B | 1.473 | 2002 |
| Novacheck | 50 | Intramuscular Psoas Lengthening Improves Dynamic Hip Function In Children With Cerebral Palsy | USA | Journal Of Pediatric Orthopaedics | 2.537 | 2002 |
| Brunner | 48 | Clinical Benefit Of Reconstruction Of Dislocated Or Subluxated Hip Joints In Patients With Spastic Cerebral-Palsy | Switzerland | Journal Of Pediatric Orthopaedics | 2.537 | 1994 |
| Perry | 48 | Electromyography Before And After Surgery For Hip Deformity In Children With Cerebral-Palsy - Comparison Of Clinical And Electromyographic Findings | USA | Journal Of Bone And Joint Surgery-American Volume | 6.558 | 1976 |
| Kim | 47 | Recurrence After Femoral Derotational Osteotomy In Cerebral Palsy | South Korea | Journal Of Pediatric Orthopaedics | 2.537 | 2005 |
| Rutz | 44 | Long-Term Results And Outcome Predictors In One-Stage Hip Reconstruction In Children With Cerebral Palsy | Switzerland | Journal Of Bone And Joint Surgery-American Volume | 6.558 | 2015 |
| Noonan | 44 | Varus Derotation Osteotomy For The Treatment Of Hip Subluxation And Dislocation In Cerebral Palsy: Statistical Analysis In 73 Hips | USA | Journal Of Pediatric Orthopaedics-Part B | 1.473 | 2001 |
| Canavese | 41 | Varus Derotation Osteotomy For The Treatment Of Hip Subluxation And Dislocation In Gmfcs Level Iii To V Patients With Unilateral Hip Involvement. Follow-Up At Skeletal Maturity | USA | Journal Of Pediatric Orthopaedics | 2.537 | 2010 |
| Aminian | 40 | Spastic Hemiplegic Cerebral Palsy And The Femoral Derotation Osteotomy: Effect At The Pelvis And Hip In The Transverse Plane During Gait | USA | Journal Of Pediatric Orthopaedics | 2.537 | 2003 |
| Huh | 39 | Surgical Management Of Hip Subluxation And Dislocation In Children With Cerebral Palsy: Isolated Vdro Or Combined Surgery? | USA | Journal Of Pediatric Orthopaedics | 2.537 | 2011 |
| Rutz | 39 | The Pediatric Lcp Hip Plate For Fixation Of Proximal Femoral Osteotomy In Cerebral Palsy And Severe Osteoporosis | Switzerland | Journal Of Pediatric Orthopaedics | 2.537 | 2010 |
| Shore | 37 | Proximal Femoral Varus Derotation Osteotomy In Children With Cerebral Palsy The Effect Of Age, Gross Motor Function Classification System Level, And Surgeon Volume On Surgical Success | USA | Journal Of Bone And Joint Surgery-American Volume | 6.558 | 2015 |

| | | | | | | |
|---|---|---|---|---|---|---|
| Schmale | 35 | High Reoperation Rates After Early Treatment Of The Subluxating Hip In Children With Spastic Cerebral Palsy | USA | Journal Of Pediatric Orthopaedics | 2.537 | 2006 |
| DiFazio | 34 | Effect Of Hip Reconstructive Surgery On Health-Related Quality Of Life Of Non-Ambulatory Children With Cerebral Palsy | USA | Journal Of Bone And Joint Surgery-American Volume | 6.558 | 2016 |
| Leet | 34 | Femoral Head Resection For Painful Hip Subluxation In Cerebral Palsy - Is Valgus Osteotomy In Conjunction With Femoral Head Resection Preferable To Proximal Femoral Head Resection And Traction? | USA | Journal Of Pediatric Orthopaedics | 2.537 | 2005 |
| Carty | 33 | The Effect Of Femoral Derotation Osteotomy On Transverse Plane Hip And Pelvic Kinematics In Children With Cerebral Palsy: A Systematic Review And Meta-Analysis | Australia | Gait & Posture | 2.746 | 2014 |
| Wren | 33 | Impact Of Gait Analysis On Correction Of Excessive Hip Internal Rotation In Ambulatory Children With Cerebral Palsy: A Randomized Controlled Trial | USA | Developmental Medicine And Child Neurology | 4.864 | 2013 |
| Schwartz | 33 | Predicting The Outcome Of Intramuscular Psoas Lengthening In Children With Cerebral Palsy Using Preoperative Gait Data And The Random Forest Algorithm | USA | Gait & Posture | 2.746 | 2013 |
| Cornell | 32 | The Hip In Children With Cerebral Palsy - Predicting The Outcome Of Soft Tissue Surgery | England | Clinical Orthopaedics And Related Research | 4.755 | 1997 |
| Dhawale | 31 | Long-Term Outcome Of Reconstruction Of The Hip In Young Children With Cerebral Palsy | USA | Bone & Joint Journal | 5.385 | 2013 |
| Knaus | 31 | Proximal Femoral Resection Arthroplasty For Patients With Cerebral Palsy And Dislocated Hips | Norway | Acta Orthopaedica | 3.925 | 2009 |
| Turker | 31 | Adductor Tenotomies In Children With Quadriplegic Cerebral Palsy: Longer Term Follow-Up | USA | Journal Of Pediatric Orthopaedics | 2.537 | 2000 |
| Abel | 31 | Asymmetric Hip Deformity And Subluxation In Cerebral Palsy: An Analysis Of Surgical Treatment | USA | Journal Of Pediatric Orthopaedics | 2.537 | 1999 |
| Valencia | 30 | Management Of Hip Deformities In Cerebral Palsy | USA | Orthopedic Clinics Of North America | 2.771 | 2010 |
| Sherk | 29 | Hip Dislocation In Cerebral-Palsy - Selection For Treatment | USA | Developmental Medicine And Child Neurology | 4.864 | 1983 |
| Wawrzuta | 28 | Hip Health At Skeletal Maturity: A Population-Based Study Of Young Adults With Cerebral Palsy | Australia | Developmental Medicine And Child Neurology | 4.864 | 2016 |
| Spiegel | 27 | Evaluation And Treatment Of Hip Dysplasia In Cerebral Palsy | USA | Orthopedic Clinics Of North America | 2.771 | 2006 |
| de Morais | 26 | Outcomes Of Correction Of Internal Hip Rotation In Patients With Spastic Cerebral Palsy Using Proximal Femoral Osteotomy | Brazil | Gait & Posture | 2.746 | 2012 |
| Krebs | 26 | Neurogenic Hip Dislocation In Cerebral Palsy: Quality Of Life And Results After Hip Reconstruction | Austria | Journal Of Childrens Orthopaedics | 2.537 | 2008 |
| Smith | 26 | Combined Adductor Transfer, Iliopsoas Release, And Proximal Hamstring Release In Cerebral-Palsy | USA | Journal Of Pediatric Orthopaedics | 2.537 | 1989 |

| | | | | | | |
|---|---|---|---|---|---|---|
| Root | 26 | Hip Adductor Transfer Compared With Adductor Tenotomy In Cerebral-Palsy | USA | Journal Of Bone And Joint Surgery-American Volume | 6.558 | 1981 |
| Boldingh | 25 | Palliative Hip Surgery In Severe Cerebral Palsy: A Systematic Review | Netherlands | Journal Of Pediatric Orthopaedics-Part B | 1.473 | 2014 |
| DiFazio | 24 | Postoperative Complications After Hip Surgery In Patients With Cerebral Palsy: A Retrospective Matched Cohort Study | USA | Journal Of Pediatric Orthopaedics | 2.537 | 2016 |
| Khalife | 24 | Risk Of Recurrent Dislocation And Avascular Necrosis After Proximal Femoral Varus Osteotomy In Children With Cerebral Palsy | Lebanon | Journal Of Pediatric Orthopaedics-Part B | 1.473 | 2010 |
| Abu-Rajab | 24 | Proximal Femoral Resection-Interposition Arthroplasty In Cerebral Palsy | Scotland | Journal Of Pediatric Orthopaedics-Part B | 1.473 | 2007 |
| Fucs | 24 | Treatment Of The Painful Chronically Dislocated And Subluxated Hip In Cerebral Palsy With Hip Arthrodesis | Brazil | Journal Of Pediatric Orthopaedics | 2.537 | 2003 |
| Mallet | 21 | One-Stage Hip Reconstruction In Children With Cerebral Palsy: Long-Term Results At Skeletal Maturity | Morocco | Journal Of Childrens Orthopaedics | 2.537 | 2014 |
| Owers | 21 | Bilateral Hip Surgery In Severe Cerebral Palsy - A Preliminary Review | England | Journal Of Bone And Joint Surgery-British Volume | 3.309 | 2001 |
| Terjesen | 20 | Adductor Tenotomy In Spastic Cerebral Palsy - A Long-Term Follow-Up Study Of 78 Patients | Norway | Acta Orthopaedica | 3.925 | 2005 |

## Table S2. Studies with citations between 10 and 20, for years 2017 to 18 October 2022

| **First author** | **Times cited** | **Article title** | **Journal** |
|---|---|---|---|
| Sung | 18 | Long-term outcomes over 10 years after femoral derotation osteotomy in ambulatory children with cerebral palsy | Gait & Posture |
| Chang | 18 | Outcomes of Isolated Varus Derotational Osteotomy in Children With Cerebral Palsy Hip Dysplasia and Predictors of Resubluxation | Journal Of Pediatric Orthopaedics |
| Kiapekos | 17 | Primary surgery to prevent hip dislocation in children with cerebral palsy in Sweden: a minimum 5-year follow-up by the national surveillance program (CPUP) | Acta Orthopaedica |
| Shore | 17 | Hip Surveillance for Children With Cerebral Palsy: A Survey of the POSNA Membership | Journal Of Pediatric Orthopaedics |
| Shore | 16 | Management Of Moderate To Severe Hip Displacement In Nonambulatory Children With Cerebral Palsy | JBJS Reviews |
| McCarthy | 15 | Establishing surgical indications for hamstring lengthening and femoral derotational osteotomy in ambulatory children with cerebral palsy | Journal Of Childrens Orthopaedics |
| El-Sobky | 15 | Bony reconstruction of hip in cerebral palsy children Gross Motor Function Classification System levels III to V: a systematic review | Journal Of Pediatric Orthopaedics-Part B |
| Church | 15 | Persistence and Recurrence Following Femoral Derotational Osteotomy in Ambulatory Children With Cerebral Palsy | Journal Of Pediatric Orthopaedics |
| Ounpuu | 15 | Long-term outcomes of external femoral derotation osteotomies in children with cerebral palsy | Gait & Posture |

| | | | |
|---|---|---|---|
| Terjesen | 14 | To what extent can soft-tissue releases improve hip displacement in cerebral palsy? A prospective population-based study of 37 children with 7 years' follow-up | Acta Orthopaedica |
| Wordie | 13 | Hip displacement and dislocation in a total population of children with cerebral palsy in Scotland | Bone & Joint Journal |
| Terjesen | 13 | Femoral and pelvic osteotomies for severe hip displacement in nonambulatory children with cerebral palsy: a prospective population-based study of 31 patients with 7 years? follow-up | Acta Orthopaedica |
| Hsieh | 11 | Guided Growth Improves Coxa Valga and Hip Subluxation in Children with Cerebral Palsy | Clinical Orthopaedics And Related Research |
| Boyer | 11 | Changes in hip abductor moment 3 or more years after femoral derotation osteotomy among individuals with cerebral palsy | Developmental Medicine And Child Neurology |
